# *GAA* compound heterozygous mutations associated with autophagic impairment cause cerebral infarction in Pompe disease

**DOI:** 10.18632/aging.102879

**Published:** 2020-03-03

**Authors:** Xiaodong Jia, Libin Shao, Chengcheng Liu, Tuanzhi Chen, Ling Peng, Yinguang Cao, Chuanchen Zhang, Xiafeng Yang, Guifeng Zhang, Jianlu Gao, Guangyi Fan, Mingliang Gu, Hongli Du, Zhangyong Xia

**Affiliations:** 1 Joint Laboratory for Translational Medicine Research, Liaocheng People's Hospital, Liaocheng 252000, Shandong, P.R. China; 2 BGI-Qingdao, BGI-Shenzhen, Qingdao 266555, P.R. China; 3 Department of Neurology, Liaocheng People's Hospital and Liaocheng Clinical School of Shandong First Medical University, Liaocheng 252000, Shandong, P.R. China; 4 Department of Clinical laboratory, Liaocheng People's Hospital and Liaocheng Clinical School of Shandong First Medical University, Liaocheng 252000, Shandong, P.R. China; 5 Department of Radiology, Liaocheng People's Hospital and Liaocheng Clinical School of Shandong First Medical University, Liaocheng 252000, Shandong, P.R. China; 6 Shandong First Medical University, Taian 271016, Shandong, P.R. China; 7 Department of Ophthalmology, Liaocheng People's Hospital and Liaocheng Clinical School of Shandong First Medical University, Liaocheng 252000, Shandong, P.R. China; 8 BGI-Shenzhen, Shenzhen 518083, P.R. China; 9 China National GeneBank, BGI-Shenzhen, Shenzhen 518120, P.R. China; 10 BGI-Fuyang, BGI-Shenzhen, Fuyang 236009, P.R. China; 11 School of Biology and Biological Engineering, South China University of Technology, Guangzhou 510006, P.R. China; 12 School of Medicine Shandong University, Jinan 250012, Shandong, P.R. China

**Keywords:** Pompe disease, cerebral infarction, GAA mutation, gut microbiome metagenomics

## Abstract

Clinical manifestations of the late-onset adult Pompe disease (glycogen storage disease type II) are heterogeneous. To identify genetic defects of a special patient population with cerebrovascular involvement as the main symptom, we performed whole-genome sequencing (WGS) analysis on a consanguineous Chinese family of total eight members including two Pompe siblings both had cerebral infarction. Two novel compound heterozygous variants were found in GAA gene: c.2238G>C in exon 16 and c.1388_1406del19 in exon 9 in the two patients. We verified the function of the two mutations in leading to defects in GAA protein expression and enzyme activity that are associated with autophagic impairment. We further performed a gut microbiome metagenomics analysis, found that the child’s gut microbiome metagenome is very similar to his mother. Our finding enriches the gene mutation spectrum of Pompe disease, and identified the association of the two new mutations with autophagy impairment. Our data also indicates that gut microbiome could be shared within Pompe patient and cohabiting family members, and the abnormal microbiome may affect the blood biochemical index. Our study also highlights the importance of deep DNA sequencing in potential clinical applications.

## INTRODUCTION

Glycogen storage disease (Pompe disease) is an autosomal recessive lysosomal storage disease caused by a deficiency of acid α-1,4-glucosidase encoded by the GAA gene (GAA [MIM: 606800] acid maltase, EC 3.2.1.20/3, 17q25.3), which is a key enzyme in hydrolyzation of lysosomal glycogen to glucose [1]. Age of onset of Pompe disease ranges from infancy to adulthood and has been classified into infantile and late-onset form. The rapidly progressive infantile-onset form is typically characterized by hypotonia, muscle weakness, motor delay, feeding problems, and respiratory insufficiency, due to a complete loss of GAA activity. The late-onset form is complicated and the patients’ symptoms are highly heterogeneous; the most common phenotype is muscle weakness. Some believe that allelic diversity underlies clinical heterogeneity in Pompe disease and a small change in residual GAA enzyme activity can profoundly affect the phenotypic expression of the disease [2–4]. Due to the relatively few reports of patients diagnosed with cerebral infarction, GAA variations related to this phenotype has barely been explored. However, in recent years, reports on the presence of Pompe disease with cerebral infarction have gradually increased [5–9]. In addition to the un-identified GAA variations for this disease subtype, one unified point is that glycogen accumulation on the inner wall of blood vessels may cause cerebral infarction, but this theory is not completely accepted [10].

**Figure 1:**
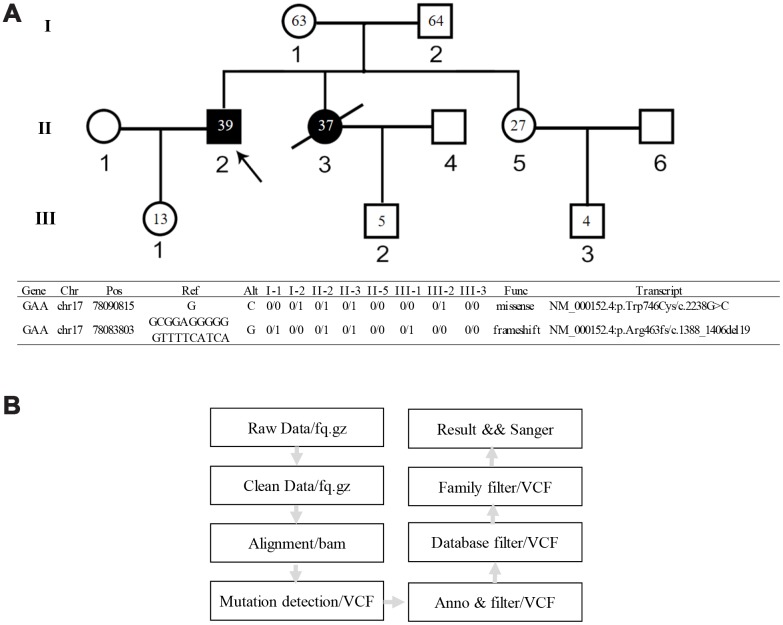
**Genetic pedigree of the family and the WGS analysis workflow.** (**A**) Genetic pedigree of the family. II-2 is the proband; II-3 is the female patient; her child III-2 has abnormal biochemical index. The age of each family member is indicated as grey. The genotype of each family member is listed in the lower panel. (**B**) Workflow of the WGS analysis.

Importance of deep DNA analysis to clarify specific phenotypes has been more and more appreciated in the medical field. The clinical utility of whole-genome sequencing (WGS) or next-generation sequencing (NGS) approaches has greatly facilitated disease diagnosis, prognosis, and prediction of targeted therapy response [11, 12]. In this study, we performed WGS on a consanguineous Chinese family of total eight members including two Pompe siblings with cerebral infarction (Figure 1A). A case study of this family has been reported in a Chinese journal in 2012. Due to the limit of sequencing technology at that time, the investigators only found a heterozygous frameshift mutation in one patient and his mother, which were insufficient to explain the genetic cause of the disease [13]. In the current study we found that both Pompe siblings had compound heterozygous mutations c.1388_1406del19 and c.2238G>C. Although the c.2238G>C mutation has been reported in many studies, to our knowledge, the new compound heterozygous mutations have not been reported. We verified the function of the two mutations in leading to defects in GAA protein expression and enzyme activity using the HEK293 cell model. Gut microbiome metagenomics analysis found that the mother's gut microbiome was very similar to that of the suspected child, and the abnormal microbiome may affect the blood biochemical indexes of the child. A year after the mother's death, the child's blood biochemical indexes returned to normal.

Finally, we present the idea on the supplement model of the pathogenesis of Pompe disease with cerebral infarction. The main viewpoint is that glycogen accumulation causes abnormal lysosomes that can't fuse with autophagosomes to form autophagic lysosomes; autophagy targets then accumulate, leading to injury to smooth muscle cells [14–16]. In our study, autophagic impairment including LC3 lipidation and p62 aggregation were verified in the HEK293 cells containing both GAA mutations. As vascular smooth muscle is the main component of the inner wall of blood vessels, its injury may change blood vessel elasticity and thickness, leading to aneurysms or ruptures.

## RESULTS

### Clinical characterization

**Figure 2:**
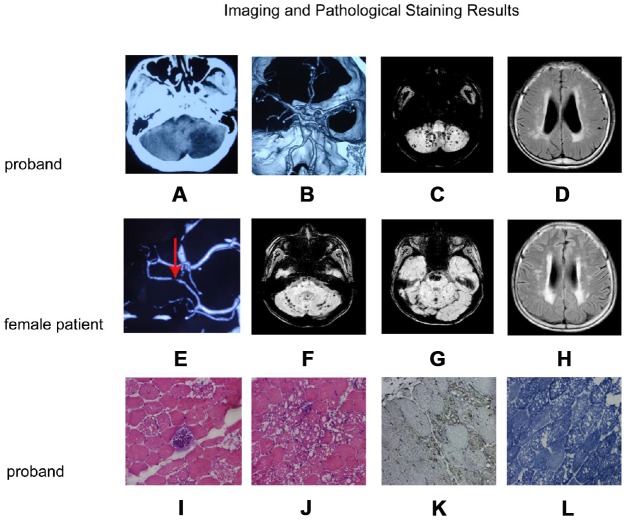
**Imaging and pathological staining results.** (**A**–**D**) and (**E**–**H**) are the imaging result of the proband and the female patient, respectively. (**I**–**L**) are the results of muscle tissue staining of the proband. (**A**) Craniocerebral CT shows left cerebellar infarction; (**B**) CTA of the brain shows a basilar artery with a fusiform aneurysm; (**C**) Cerebral MRI SWI shows multiple bleeding focus in both hemispheres of the cerebellum; (**D**) MRI T_2_FLAIR images shows multiple ischemic lesions in both lateral ventricles and deep white matter; (**E**) CTA of the brain shows a localized stenosis of the right posterior cerebral artery; (**F**, **G**): MRI SWI shows recurrent cerebellopontine hematoma and multiple micro-hemorrhagic foci of cerebellum and brainstem; (**H**) Craniocerebral MRI T_2_FLAIR images shows multiple ischemic lesions in both lateral ventricles and deep white matter; (**I**, **J**): H&E staining muscle fibers of proband were slightly different in size, polygonal in shape, and slightly increased in kernel fibers. (**K**) LAMP2 staining was enhanced in the vacuolar muscle fiber, and the distribution was significant at the margin of vacuolar muscle fiber. (**L**) NADH staining showed the interphase distribution of two types of fibers, the mesh-like structure in vacuolar fibers was disordered, and the activity of NADH in vacuolar region was absent.

The proband was hospitalized for persistent dizziness and unsteady gait. At the age of 14, the patient started to develop weakness in both legs and complained constant fatigue. Examination revealed that the patient has normal intelligence, no facial palsy, scoliosis, instability in the finger-nose test, and muscle atrophy throughout the body. Craniocerebral CT showed left cerebellar cerebral infarction (Figure 2A); cerebral MRI SWI shows multiple microhemorrhage loci in both hemispheres (Figure 2C); MRI T_2_FLAIR showed multiple ischemic lesions in both lateral ventricles and deep white matter (Figure 2D). Computed tomography angiography (CTA) showed bilateral acute curvature of the internal carotid artery (C2 segment); the left side was significant. Calcified plaques were formed in bilateral vertebral artery and the lumen exhibited segmental stenosis. Multiple bilateral local stenoses occurred in the P1-P2 segments of the posterior cerebral arteries. Basilar artery aneurysm was observed (Figure 2B). Electromyography revealed myogenic damage. The patient’s hematological examination readings were: alanine aminotransferase (ALT) 39 IU/L, aspartate aminotransferase (AST) 132 IU/L, creatine kinase (CK) 325 IU/L, and lactate dehydrogenase (LDH) 180 IU/L.

H&E staining showed that the patient’s muscle fibers were slightly different in size, polygonal in shape, and slightly increased in kernel fibers. A large number of muscle fibers were seen in the submucosal and intramuscular area with irregular vacuoles, and some muscle fibers were almost completely vacuolated. Occasionally, basophilic granules deposition were seen between the muscle fibers. No necrosis accompanied by phagocytosis and regenerated fibers were observed. No focal infiltration of inflammatory cells in the muscle interstitium, and there was a slight hyperplasia of the muscle inner membrane (Figure 2I, 2J). Lysosomal Associated Membrane Protein 2 (LAMP2) immunohistochemical staining was enhanced in the vacuolar muscle fiber, and the distribution was enriched at the margin of vacuolar muscle fiber (Figure 2K). Coenzyme I (NADH) staining showed the interphase distribution of two types of fibers, the mesh-like structure in vacuolar fibers was disordered, and the activity of NADH in vacuolar region was absent (Figure 2L). No increase of lipid droplets was observed by OR0 staining. Electron microscopy was used to examine muscle fibril structure; myofibril and muscle glycogen particles increased significantly under the plasma membrane. We also observed focal myofibril destruction, glycogen granules, cystic structure, and lysosomes in addition to the phenomenon of many membranous vacuoles full of glycogen particles. A large number of mitochondria accumulated near the nuclear membrane but we did not see lattice-form inclusion bodies in the mitochondria. Another phenomenon was that mesenchymal cells were visible within the glycogen accumulation.

The female patient, the younger sister of the proband, died in December 2017 from respiratory failure caused by a lung infection. Her intelligence was normal, and her left nasolabial groove was shallow; she exhibited whole-body muscle atrophy and left finger-nose test instability. Electrocardiogram showed a decreased ST segment. She had ruptured mitral tendinous cord and valve prolapse. Her mitral valve was thickened and with neoplasm. Her liver and spleen were large. MRI susceptibility-weighted imaging (SWI) showed recurrent cerebellopontine old hemorrhagic focus and multiple micro-hemorrhagic foci in cerebellum and brainstem (Figure 2F, 2G). Multiple patchy abnormal signals were detected in the brain stem, bilateral brain semi-oval center, radiation crown area, and left sub frontal cortex. Craniocerebral MRI T_2_/FLAIR images shows multiple ischemic lesions in para lateral ventricles and white matter (Figure 2H). CTA of the brain showed stenosis at the left anterior cerebral artery and the right posterior cerebral artery (Figure 2E). Electromyography revealed myogenic damage. The patient’s hematological examination readings were: ALT 71 IU/L, AST 280 IU/L, CK 484 IU/L, and LDH 375 IU/L.

The child of the female patient appeared normal. However, some abnormal blood biochemical indexes were observed during the physical examination, including: ALT 14 IU/L, AST 48 IU/L, CK 556 IU/L, LDH 246 IU/L, which led to the suspicion that the child may be a Pompe disease patient. A year after the mother's death, the child's biochemical index became normal, the hematological examination readings were: ALT 14 IU/L, AST 31 IU/L, CK 97 IU/L, and LDH 225 IU/L.

### Compound heterozygous mutations of GAA gene

**Figure 3:**
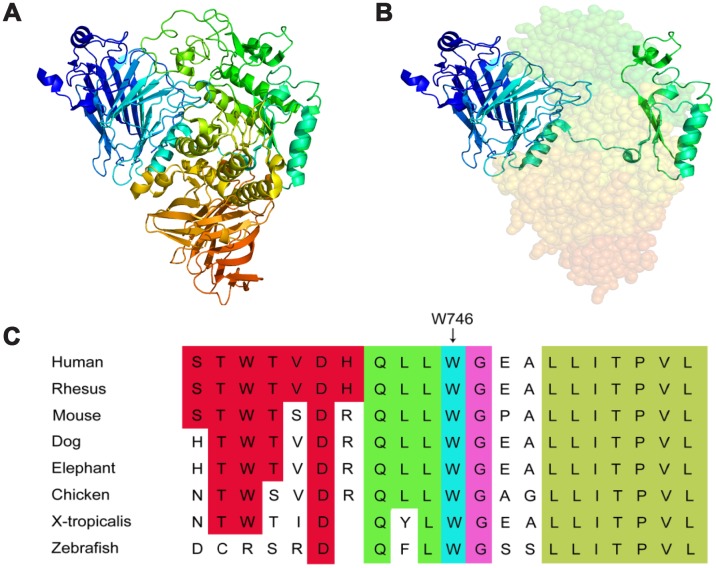
**3D structure of lysosomal alpha-glucosidase and conservation of the missense mutation among different species.** (**A**) the normal 3D structure of lysosomal alpha-glucosidase. (**B**) the 3D structure of lysosomal alpha-glucosidase resulting from the frameshift mutation, shaded part can not expression because of premature translation termination. (**C**) the missense mutation reported in this study is highlighted by a blue rectangle, illustrating that the p.Trp746Cys mutation is in a highly conserved region.

**Figure 4:**
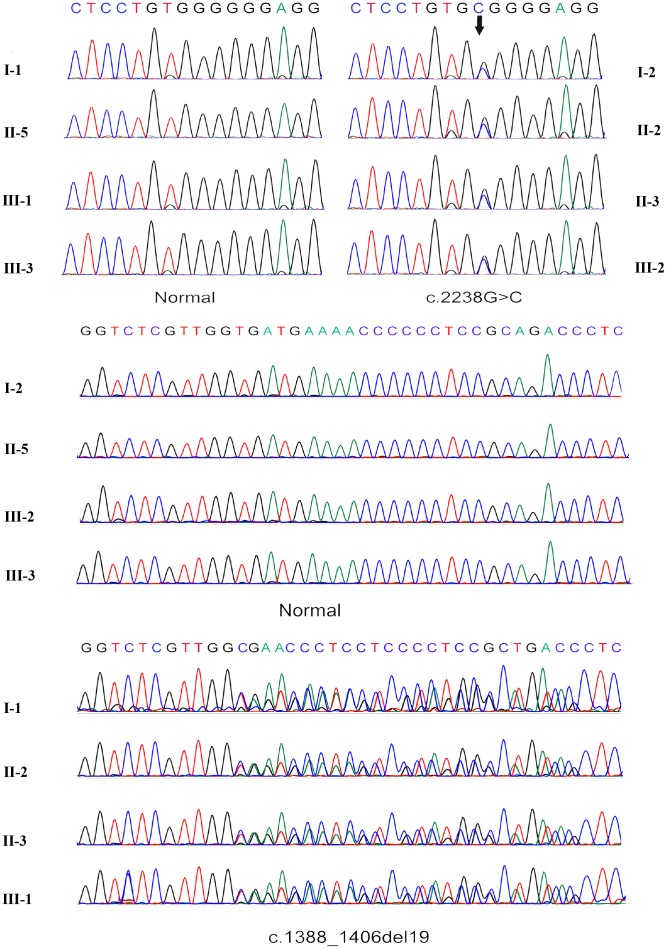
**Sanger sequencing verification of the c.2238G>C mutation and frameshift mutation c.1388_1406del19.**

WGS data from all subjects were thoroughly analyzed (Figure 1B). In comparing with the normal family members, both patients beard two GAA compound heterozygous mutations, one was a missense mutation c.2238G>C (p.Trp746Cys) from the father and the other was a frameshift mutation c.1388_1406del19 (p. Arg463fs) from the mother. The first mutation c.2238G>C (p.Trp746Cys) in exon 16 causes a change from nonpolar aromatic tryptophan to polar aliphatic cysteine at codon 746 which is in a highly conserved region (Figure 3C) and has been reported to affect the enzymatic function of acid α-glucosidase [5, 17]. The second mutation c.1388_1406del19 (p. Arg463fs) in exon 9 was a frameshift mutation caused by a deletion of 19 bases. This mutation leads to premature termination of protein translation; a frameshift mutation occurs once the deletion takes place, which results in an unexpected stop to translation at amino acid 462 (Figure 3A, 3B). The truncated polypeptide chain cannot maintain its normal conformation, so the protein is unable to function normally. In addition to the above single nucleotide polymorphisms (SNP) and indel, c.1726G>A and c.2065G>A mutation sites from the father GAA genome and c.2446G>A from the mother GAA genome were also found. These three mutation sites have been reported and have racial heterogeneity, c.1726G>A and c.2065G>A are mainly distributed in Asian populations, with higher frequency in China, Taiwan, and Japan, c.1726G>A mutation can lead to a decreased enzyme activity, but this mutation does not cause disease; mutation of c.2446G>A also has a higher allele mutation frequency in the population [4, 18, 19]. So, in this study, the compound heterozygous mutations c.2238G>C and c.1388_1406del19 of GAA gene lead to familial glycogen storage disease type II. For the child of the female patient, we only found the c.2238G>C mutation. The other chromosome was normal; therefore, we can rule out the possibility of the child to develop the disease. We performed Sanger sequencing on these two pathogenic sites in all the members, and the results of Sanger validation were consistent with the whole-genome analysis (Figure 4).

### Analysis of genetic risk locus and autophagy-related gene SNPs associated with ischemia/hemorrhagic stroke and cardiovascular diseases

In addition to GAA gene, we examined variations of two panels of genes to explore the mechanism behind the cerebral infarction of the patients. One is 15 genome-wide risk loci associated with ischemia and hemorrhagic stroke [20]; the other is 7 autophagy genes with the most significant association with cardiovascular disease [21]. The risk allele frequencies for these loci are highly identified in big cohort studies. In our studies, 7 out of 15 risk alleles of hemorrhagic stroke-associated genes were examined in all family members including the 2 Pompe patients and 6 normal members (Supplementary Table 1). Considering each individual risk allele has 2-66% frequency in the general population [20], all 8 family members showed more than 50% of the risk alleles may indicate high genetic risk with the disease. For autophagy genes associated with cardiovascular disease, 2 out of 7 risk alleles were examined in the family, both appear in the 2 Pompe patients, and 6/6 or 3/6 in the normal members (Supplementary Table 2).

### GAA activity in patient samples

GAA activity of the proband and the child measured by peripheral blood lymphocytes enzyme activity method were 3.77 nmol/h/mg and 9.55 nmol/h/mg (reference value 14 nmol/h/mg), respectively. In general, the residual GAA activity in patients was 1%~30%. Although the child's enzyme activity was slightly below normal, it was still in the normal range. There was no GAA activity result for the female patient because she passed away.

### Functional characterization of GAA mutations

**Figure 5:**
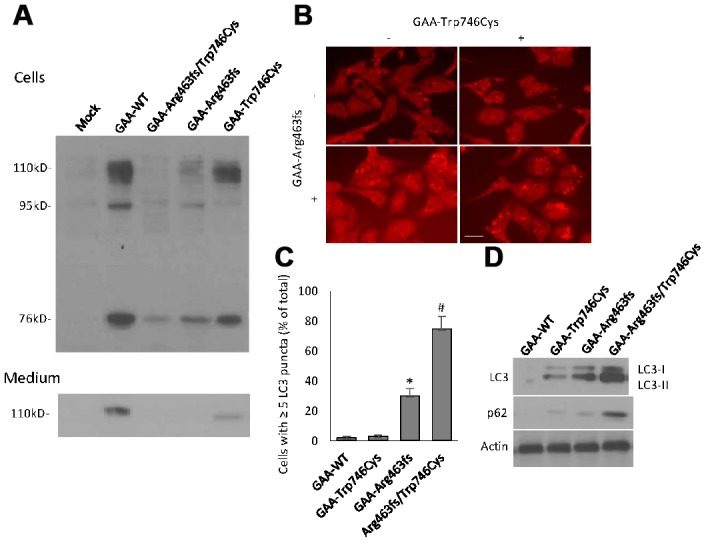
**GAA expression and autophagy induction in transiently expressed HEK293 cells.** (**A**) Western blot analysis of GAA protein expression in cells and culture medium that were harvested at 48 hour after transfection. Different molecular forms of GAA protein, i.e., 110kD precursor, 95 kD partially processed intermediate and 76 kD mature GAA were separated by SDS-PAGE and visualized by immunoblotting. Top panel: cell lysates; bottom panel: culture media. (**B**) Representative immunofluorescent images of the LC3-positive autophagic puncta in HEK293 cells with different transfection. Scale bar: 10μm. (**C**) Quantification of the % of cells contains ≥ 5 LC3 puncta. (**D**) Western blot analysis of LC3 and p62 in transfected cells.

HEK-293 cells constitutively express human GAA which is considered as a background signal (Figure 5A). Cells transfected with the GAA wild type construct (GAA-WT) contained 3 forms of GAA molecular species, i.e., the 110 kD precursor, the 95 kD partially processed intermediate and the 76 kD mature GAA. The medium contained only the 110 kD GAA precursor (Figure 5A). The quality and quantity of different molecular species of GAA protein were evaluated according to the previous reported criteria [22]. Although all 3 forms of GAA were detected in either of the GAA-Trp746Cys or GAA-Arg463fs transfected cells, the quantity of each decreased moderately with GAA-Trp746Cys mutation and severely with GAA-Arg463fs mutation (Table 1). In the cells transfected with both constructs, all 3 forms of GAA were barely detected (Table 1). Mutation severity was evaluated using the scoring system based on the GAA activity in the medium and in the cells [22]. Results in Table 1 indicated that the combination of GAA-Trp746Cys and GAA-Arg463fs mutations led to a “class B—potentially severe” deficiency of GAA activity.

**Table 1:** GAA protein and activity variation in transient transfected HEK293 cells.

	**M110**	**C110**	**C95**	**C76**	**M%**	**C%**	**Class**
GAA-Trp746Cys	3,4	3,4	3,4	3,4	6.4	28.5	D
GAA-Arg463fs	1,1	3,4	3,4	3,4	1.1	8.3	C
GAA-Trp746Cys/ Arg463fs	1,1	2,4	2,4	2,4	0.5	2.5	B

Note: M110, C10, C95, and C76 stand for the various molecular forms of GAA during posttranslational modification and were visualized by western blot in Figure 5A. The numbers refer to the severity scaling [22]. Class A mutations are very severe, class B mutations are potentially less severe, class C mutations are mild, and class D mutations are probably nonpathogenic. M% stands for the percentage of GAA activity in the culture medium and C% for the percentage of GAA activity in the cells as compared to wild-type GAA activity.

Autophagy impairment was examined in the GAA constructs-transfected HEK293 cells after culturing with glucose-free medium for 24-hour. Morphological analyses showed the presence of ≥5 LC3 puncta in the vast majority of cells with the double GAA-Trp746Cys and GAA-Arg463fs transfection (Figure 5B, 5C). Protein expressions of LC3 and p62, two markers of autophagosome formation and clearance, respectively, were quantified by western blot analysis (Figure 5D). GAA-Trp746Cys and Arg463fs transfected cells had a massive increase in lipidated LC3 and p62 protein, indicating a autophagy failure.

### Analysis of gut microbiome metagenomics

**Figure 6:**
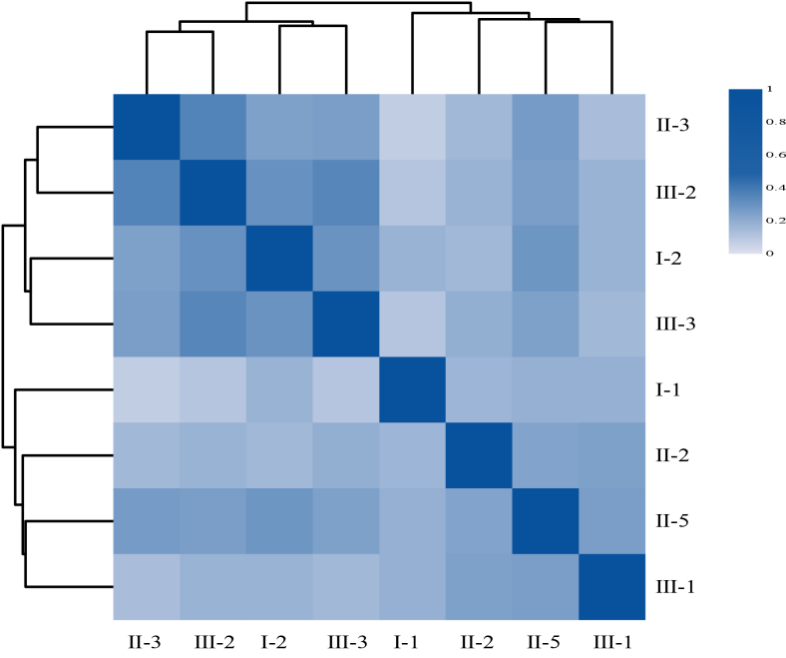
**The heatmap of Person’s correlation between members of the Family according to their respective gene abundance.** Rows and columns represent individuals, on the left is the clustering of the sample. Different colors reflect the corresponding correlation coefficient.

We tried to explore why the child had abnormal blood biochemical index when he lived with his Pompe mom. Because intestinal flora has been shown to be associated with a variety of chronic diseases [23–25] and cohabiting family members normally share microbiota with one another [26, 27], we extracted DNAs from the fecal samples of the family members and performed microbiome metagenomics analysis. We generated 111.15Gb raw intestinal metagenomic data and 102.33 Gb high quality data after removing adaptor sequences, low quality data, and host data (Table 2). An average of 12.79Gb high quality data was obtained for each sample. Subsequent analysis found that II-3 (the female patient) has the lowest alpha diversity among the three diversity indices, compared to other individuals of the family (Table 3), indicating her intestinal flora was disordered. Her child’s (III-2) gut flora composition is similar to hers (Table 3), with the second lowest alpha diversity. Specifically, the correlation analysis shows that II-3 and III-2 has the highest correlation coefficient (Figure 6) according to their respective gene abundance, suggesting that the mother may have a certain influence on the child's intestinal flora [28]. Furthermore, we identified several differential species such as *Actinobacillus, Histophilus, Mobiluncus,* and *Thermoanaerobacterium* between patients and normal family members, however, due to the limited number of samples, we could not conclude any practical significance.

**Table 2:** Statistic analysis of the raw sequencing data and clean data.

**Sample**	**Raw_reads**	**Clean_reads**	**GC_rate(%)**	**Q20(%)**	**Q30(%)**	**Rate(%)**
I-1	144,423,710	133,047,782	46.63	95.31	85.19	92.12
I-2	124,706,334	114,120,378	46.47	94.81	83.80	91.51
II-2	151,862,788	140,826,868	46.35	95.67	86.15	92.73
II-3	106,989,394	98,095,844	44.92	95.03	84.39	91.69
II-5	171,312,630	159,937,778	46.72	95.91	86.77	93.36
III-1	150,264,330	139,860,546	47.95	95.79	86.52	93.08
III-2	128,088,996	118,490,886	47.43	95.46	85.64	92.51
III-3	133,805,476	123,609,822	48.41	95.61	86.07	92.38

Note: Paired-end reads were generated with BGISEQ-500 platform, then the reads with sequencing adapters, N base, poly base, low quality etc. were filtered out with SOAPnuke.

**Table 3:** Alpha diversity of the gut microbiome metagenomics.

**Sample**	**shannon**	**chao1**	**Gene number**
I-1	17.00	1,012,086	842,437
I-2	17.50	1,558,589	1,250,094
II-2	16.31	1,205,269	920,298
II-3	15.79	748,586	564,231
II-5	16.05	1,254,924	986,863
III-1	17.89	1,545,801	1,289,158
III-2	16.37	875,557	701,548
III-3	16.86	983,039	812,747

Note: Shannon, chao1 and gene number are three different calculation indicators for the diversity analysis. chao1 and Gene number index can reflect the species richness of the community, whereas Shannon index can reflect the species diversity of the community, considering both species richness and evenness.

## DISCUSSION

In this study, novel compound heterozygous mutations that cause Pompe disease with cerebral infarction were reported, i.e., missense mutation c.2238G>C and frameshift mutation c.1388_1406del19. Compared with missense mutation, the consequence of frameshift mutation was more serious, almost complete loss of enzyme function (Figure 5 and Table 1). Mutation of c.2238G>C was reported to be more common in Asian populations. Wan et al. has reported a homozygous c.2238G>C mutation in a juvenile onset patient with other two heterozygous mutations [29]. A study on the largest series of mainland Chinese late-onset Pompe patients, including 27 patients from 24 mutationally unrelated families showed that the majority of patients (15/27, 55.56%) carried the c.2238G>C mutation of GAA, and that the allele frequency of c.2238G>C was as high as 27.08%, making it the most common mutation in this group [30]. Zhang et al. identified a compound heterozygous mutation involving exon 4 (c.827-845del19) and exon 16 (c.2238G>C) in a Chinese female patient [5]. According to these reports and the results of our study, we postulate that higher mutation frequency of c. 2238 G>C in Chinese and Asian people is a common genetic basis of late-onset Pompe disease. The phenotype caused by this mutation is not serious, but most of the late-onset Pompe disease patients have compound heterozygous mutations, and the combination of two different mutations leads to the more serious phenotypes. Indeed, compound heterozygous mutations that occur on different copies of genes are reported to completely "knock-out" gene function [31]. In the case of cerebrovascular involvement as the severe symptom, the c.2238G>C and c.1388_1406del19 compound heterozygote may be the disease-leading genetic defect.

The pathogenesis of Pompe disease is not as simple as glycogen accumulation, and the root cause has been proposed as abnormal autophagy. Studies on GAA knockout mice (KO) and the efficacy of enzyme replacement therapy have proved that autophagy defects are a main pathogenesis of Pompe disease [18, 32–35]. According to the reports, overexpression of modulators of transcription factor EB (TFEB) in cultured myoblasts from a Pompe disease murine model reduced glycogen store and lysosomal size, facilitated autophagosome processing, and alleviated excessive accumulation of autophagic vacuoles [18, 36]. In our study, we specially paid attention to the autophagy-related genes that have been associated with cardiovascular diseases [21] when performing the WGS analysis. We did find 2 variations in this gene list (Supplementary Table 2) but could not claim their significance. Furthermore, we even could not rule out the transcriptional or post-translational change that may happen to the autophagy-related genes during disease development. In our *in vitro* experiments, we demonstrated that the compound heterozygous mutations indeed induced autophagy impairment in HEK293 cells. Morphologically autophagosome accumulation of LC3 positive puncta were seen in ~80% cells carrying both mutations; biochemically the protein expression of LC3 and p62 increased significantly after 24-hour glucose deprivation. These results may indicate that glycogen accumulation and autophagy deficiency are both caused by the identified compound heterozygous mutations and they are the pathogenic mechanism of the cerebral infarction subtype of the disease.

In recent years, there have been many reports about the presence of Pompe disease with basal aneurysm, as well as cases related to arterial abnormality [5–9]*.* To exclude the existence of co-occurrence genetic risk factors, we examined the genome-wide risk loci for ischemic and hemorrhagic stroke [20], and found 7 risk alleles in all family members (Supplementary Table 1). Although there was no other ischemia or stroke history in this family except the two Pompe disease siblings, we could not exclude these risk factors. Based on these, we considered that late-onset Pompe disease with cerebral infarction is caused by multiple and complex genetic factors, and may also be associated with autophagy accumulation. Recent work shows that age-dependent autophagic buildup in the GAA gene knockout mice is due to the progressive nature of the disease [33]. This suggests that as the disease progresses, the autophagy accumulation can become severe [10]. A growing number of studies have shown that autophagy was associated with atherosclerosis and aneurysm formation, and that autophagy deficiency within vascular smooth muscle causes cell death, as well as aneurysm rupture [19, 37]. Although an animal study using Pompe model is not performed to verify the *in vivo* function of the heterozygous mutations in autophagy deficiency and cerebral infarction, the *in vitro* study provided fairly support to speculate that autophagy impairment in blood vessel smooth muscle cells or endothelial cells are involved in the formation of basal aneurysm and vascular remodeling [38, 39]. We hope that this result will guide clinicians to increase brain screening for Pompe patients, and even to extend the examination of cardiovascular and renal vessels, which is of great significance for monitoring disease progression and studying the relationship between phenotype and genotype of late-onset Pompe disease.

In addition, another reason to study the family again was that the child of the female patient was found to have abnormal blood biochemical indicators during physical examination. The GAA genetic screen ensures that the child is just a carrier of one pathogenic gene but not with Pompe disease. In order to explain the phenomenon of the child’s abnormal biochemical index, we studied the gut microbiome of the patients and their families. As far as we know, metagenomic studies in the field of family genetic disease have barely been reported. Although our work did not achieve statistical significance with limited sample size, it’s still noteworthy to report the results that the mother patient could share her abnormal gut microbe with her child and the abnormal microbe could affect the body metabolism. It has been reported that the intestinal microbiome of normal newborn is mainly from the mother, and breastfeeding also affects the intestinal microbiome of the baby. As the child grows, the intestinal microbiome will tend to be stable [40]. We believed that the mother patient’s childbirth and breastfeeding delivered her gut microbe to the child then interfere with the biochemical indicators of the child. At this stage, we couldn’t make any solid conclusion from the metagenomic studies, however, this is a general problem for all rare diseases. At least our preliminary study suggested that metagenomics can explain inconsistencies between disease genotype and clinical phenotype, which may be useful in clinic for excluding a suspected diagnose of metabolic-related disease.

## MATERIALS AND METHODS

### Patients and controls

Two sibling patients, clinically diagnosed with Pompe disease, were recruited from Liaocheng People's Hospital Department of Neurology. Additionally, a 5-year-old boy, the female patient’s child, had suspected symptoms and had been mis-diagnosed with Pompe disease. Six healthy subjects from this family were selected as controls, including the parents of the patients, children of the patients, sister of the patients, and the spouse of the female patient (Figure 1).

The study conformed to the tenets of the Declaration of Helsinki and was approved by the ethics committee of Liaocheng People's Hospital, Shandong province. Informed consent was obtained from all patients and their families.

### Whole-genome sequencing and data analysis

Peripheral blood samples were obtained from the sibling patients and their family members. Genomic DNA was extracted from peripheral blood using standard protocols. The DNA samples were sequenced by WGS on a BGISEQ-500 platform for paired-end 100 bp reads. The average sequencing depth ranged from 40.54 to 44.12X.

Low quality reads were filtered by SOAPnuke, then mapped against UCSC hg19 (http://genome.ucsc.edu/) by BWA (http://bio-bwa.sourceforge.net/). The SNPs and indels were detected by GATK. Single nucleotide variants and indels were filtered using the following criteria: (i) homozygous or compound heterozygous; (ii) absent or with a minor allele frequency value <0.01 in public databases [including dbSNP, 1000 Genomes Project, the NHLBI Exome Sequencing Project (ESP), the Exome Aggregation Consortium (ExAC) and gnomAD]; (iii) exclude non-coding regions variant, intergenic region variant, downstream variant, upstream variant, intron variant, synonymous variant, 3-prime UTR variant, 5-prime UTR variant; and (iv) possible pathogenic effects of the identified variant were predicted by SIFT (https://sift.bii.a-star.edu.sg/) and PolyPhen-2 (http://genetics.bwh.harvard.edu/pph2/). Sanger sequencing was used to validate the novel compound heterozygous mutations in *GAA* gene identified by the WGS.

### GAA activity assays

GAA activity in peripheral white blood cells was measured using a standard fluorescence assay. A synthetic substrate 4-mug was used, which when hydrolyzed by GAA releases a fluorophore. Acarbose was used to inhibit its isoenzyme, and the peripheral white blood cells were detected by the Molecular Devices SpectraMax Gemini XPS. White blood cell homogenate was prepared by ultrasound and protein concentration was determined by the BCA method. Enzyme activity measurement mixture included: 50mL cell homogenate, 10μL 100μM acarbose, and 50μL 6mM 4-methyl ketone of umbrella-α-D-pyran glycosidase fluid. After a 1 hr incubation in a 37°C water bath, 1.25 mL glycine carbonate buffer (pH=10.3, 0.17 M) was added to terminate the reaction. Gemini XPS was used to measure the fluorescence intensity; each group required a blank control.

### Functional analysis of the GAA mutations

A pSHAG2 expression vector containing the wild-type GAA open reading frame (GAA-WT) was used to generate site-directed mutagenesis. The constructs of missense mutation c.2238G>C (GAA-Trp746Cys) and frameshift mutation c.1388_1406del19 (GAA-Arg463fs) were introduced in the plasmid individually or simultaneously by using the QuickChange® Site-Directed Mutagenesis Kit (Agilent Technologies Inc.). The integrities of the mutant constructs were confirmed by direct sequencing. The constructs were transduced into HEK293 cells when 80–90% of confluence at 1.5 mg of GAA-WT or mutant constructs using transient transfection reagent Effectene (Qiagen). Mock transfected cells served as negative controls. Cells were harvested at 48-hour with lysis buffer (50 mM Tris–HCl pH 7.0, 150 mM NaCl, 50 mM NaF, and 1% TritonX-100), centrifuged at 10,000 g for 10 min, then the supernatant fraction was recovered. GAA activity was measured in both medium and cell homogenates. Mutation severity was evaluated based on the GAA activity in the medium and in the cells, and on the quality and quantity of the different molecular species of GAA proteins [22]. Cell lysate and immunoprecipitated GAA from the medium were subjected to SDS-PAGE and Western-blotting analysis using GAA specific polyclonal rabbit antibody (SAB2100872, Sigma Aldrich) and goat anti-rabbit secondary antibody.

### Examination of autophagy deficiency *in vitro*

HEK293 cells with different GAA constructs were cultured in glucose-free medium for 24-hour before analyzing autophagy. LC3 Antibody Kit for Autophagy (L10382, Thermo Fisher Scientific Inc) was used to stain HEK293 cells and fluorescent images were taken by Olympus FV1000 confocal microscope. Cells with ≥5 LC3 puncta were quantified manually by two independent research staff in a single-blind way. Protein expressions of LC3 (LC3 I and II) and p62 were quantified by western blot analysis using rabbit anti-LC3 and anti-p62 primary antibodies (#2775 and #5114, Cell Signaling Technology Inc).

### Gut microbiome metagenomic sequencing and data processing

Fecal samples were collected from all 8 family individuals and then frozen immediately at -80°C before transportation to the laboratory for DNA extraction. Subjects did not receive any antibiotic treatment and did not take any probiotics for at least one month before sample collection. DNA was extracted using the standard protocol. All samples were sequenced on a BGISEQ-500 platform with paired-end 100 bp reads, and filtered to remove adaptor contamination, low quality reads, and host data (hg19) by SOAPnuke. The remaining high quality data was aligned to IGC to profile gene abundance by bowtie2, and the species abundance and the functional abundance were summarized from their respective genes [41]. Differential genera were determined using the function of t-test in R. The alpha diversity was analyzed with the QIIME program. The Pearson coefficient was used to calculate the relationship between family individuals according to their gene abundance.

### Accession numbers

The sequence data has been deposited in the CNSA databases under the following accession numbers: CNP0000237.

## Supplementary Material

Supplementary Tables
