# Supplementary material for: GAA compound heterozygous mutations associated with autophagic impairment cause cerebral infarction in Pompe disease
Source: Aging (Albany NY). 2020 Mar 3;12(5):4268–82. doi: 10.18632/aging.102879 (PMC7093195; doi:10.18632/aging.102879)
Supplement: Supplementary Tables [file aging-12-102879-s001..pdf]

SUPPLEMENTARY TABLES

Supplementary Table 1. Highly frequent SNPs associated with ischemic and hemorrhagic stroke are not differentially examined in the Pompe patients and their family members.

Gene	Lead-SNP	Chromosome	Position	Allele	Pompe patients	Normal members
ABO	rs505922	9	136149229	T>C	2/2	5/6
APOE	rs429358	19	45411941	T>C	0/2	0/6
AQP9	rs4471613	15	58551694	G>A	0/2	0/6
CDC5L	rs556621	6	44594159	T>G	2/2	4/6
FOXF2	rs12204590	6	1337393	T>A	0/2	0/6
HABP2	rs11196288	10	115057443	A>G	2/2	6/6
HDAC9	rs2107595	7	19049388	G>A	2/2	5/6
MMP12	rs660599	11	102729757	G>A	0/2	0/6
NINJ2	rs11833579	12	775199	G>A	1/2	5/6
PITX2	rs6843082	4	111718067	G>A	0/2	2/6
PMF1	rs2984613	1	156197380	C>T	2/2	5/6
PRKCH	rs2230500	14	61924239	G>A	0/2	1/6
SH2B3/ALDH2	rs10744777	12	112233018	T>C	2/2	6/6
TSPAN2	rs12122341	1	115655690	C>G	0/2	0/6
ZFHX3	rs879324	16	73068678	G>A	0/2	1/6

The frequency of these SNPs were identified from several independent big cohorts in the review article [20].

Supplementary Table 2. Common autophagy-related gene SNPs associated with cardiovascular diseses are not differentially examined in the Pompe patients and their family members.

Gene	Lead-SNP	chromosome	Position	Allele	Pompe patients	Normal members
ATG7	rs2447607	chr3	11552027	C>T	2/2	6/6
ATG4D	rs7255312	chr19	10663165	C>T	2/2	3/6
ATG4C	rs6587988	chr1	63252766	C>T	0/2	0/6
AMBRA1	rs11038913	chr11	46559730	T>C	0/2	0/6
ATG13	rs8914	chr11	46699124	G>A	0/2	0/6
ATG16L1	rs4944804	chr11	72520530	C>A	0/2	0/6
MAP1LC3A	rs6088521	chr20	33133184	A>C	0/2	1/6

The frequency of these SNPs were identified in the research article [21].
